# 1774. Surveillance for Emerging and Reemerging Pathogens Using Pathogen Agnostic Metagenomic Sequencing in the United States: A Critical Role for Federal Government Agencies

**DOI:** 10.1093/ofid/ofad500.1603

**Published:** 2023-11-27

**Authors:** Diane L Downie, Preetika Rao, Corinne David-Ferdon, Sean Courtney, Justin Lee, Claire Quiner, Pia MacDonald, Keegan Barnes, Shelby S Fisher, Joanne D Andreadis, Jasmine Chaitram, Matthew R Mauldin, Reyolds M Salerno, Jarad Schiffer, Adi Gundlapalli

**Affiliations:** U.S. Centers for Disease Control and Prevention, Atlanta, Georgia; U.S. Centers for Disease Control and Prevention, Atlanta, Georgia; U.S. Centers for Disease Control and Prevention, Atlanta, Georgia; U.S. Centers for Disease Control and Prevention, Atlanta, Georgia; U.S. Centers for Disease Control and Prevention, Atlanta, Georgia; RTI International, Research Triangle Park, North Carolina; RTI International, Research Triangle Park, North Carolina; RTI International, Research Triangle Park, North Carolina; RTI International, Research Triangle Park, North Carolina; U.S. Centers for Disease Control and Prevention, Atlanta, Georgia; U.S. Centers for Disease Control and Prevention, Atlanta, Georgia; U.S. Centers for Disease Control and Prevention, Atlanta, Georgia; U.S. Centers for Disease Control and Prevention, Atlanta, Georgia; Centers for Disease Control and Prevention, Atlanta, Georgia; U.S. Centers for Disease Control and Prevention, Atlanta, Georgia

## Abstract

**Background:**

The surveillance and identification of emerging, reemerging, and unknown infectious disease pathogens is essential to national public health preparedness and relies on fluidity, coordination, and interconnectivity between public and private surveillance systems and networks. Implementing and sustaining programs to detect emerging and re-emerging pathogens in humans using advanced molecular methods, such as metagenomic sequencing, requires large investments in testing equipment and developing networks of clinicians, laboratory scientists, and bioinformaticians.

**Methods:**

This study sought to gain an understanding of the role of federal government agencies in supporting such pathogen-agnostic testing of human specimens in the United States. A landscape analysis was conducted of federal agency websites for publicly accessible information on the availability and type of pathogen-agnostic testing and details on flow of clinical specimens and data. The website analysis was supplemented by expert review of results with representatives from the federal agencies.

**Results:**

Operating divisions within the Department of Health and Human Services and the US Department of Veterans Affairs have developed and sustained extensive clinical and research networks to obtain patient specimens and perform metagenomic sequencing at scale. Metagenomic facilities supported by US agencies were not equally geographically distributed across the US. Although many entities have work dedicated to metagenomics and/or support emerging infectious disease surveillance specimen collection, there was minimal apparent collaboration across agencies.

**Conclusion:**

Developing a national sentinel surveillance network with existing resources and infrastructure could potentially increase efficiency, accelerate identification of emerging biothreats, and support coordinated intervention strategies that reduce morbidity and mortality.

**Disclosures:**

**All Authors**: No reported disclosures

